# A 58-Year Old Male with Cognitive Deteriorations Caused by Septum Pellucidum Cyst: A Case Report

**DOI:** 10.3389/fnagi.2017.00299

**Published:** 2017-09-13

**Authors:** Li-Ming Chen, Ming-Xin Zhu, Yu-Fen Zhang, Se-Hui Ma, Yao Yi, Lie-Xin Xia, Yan Wu, Lei Pei

**Affiliations:** ^1^Department of Neurology of the First People’s Hospital of Jingzhou (The First Affiliated Hospital of Yangtze University) Jingzhou, China; ^2^Department of Neurosurgery, Tongji Hospital, Tongji Medical College, Huazhong University of Science and Technology Wuhan, China; ^3^Department of Neurobiology, School of Basic Medicine, Tongji Medical College, Huazhong University of Science and Technology Wuhan, China; ^4^Department of Neurology, Union Hospital, Tongji Medical College, Huazhong University of Science and Technology Wuhan, China

**Keywords:** septum pellucidum cyst, cognitive impairment, mini-mental state exam, montreal cognitive assessment, dementia, neuroendoscopy

## Abstract

Dementia is known to be induced by vascular dementia and certain neurodegenerative diseases. The presenting features of disordered memory, intellect and personality often result in referral to a neurologist initially. Septum pellucidum cyst (SPC) is a rare clinical finding and defined as a cystic structure between the lateral ventricles. SPC induced memory disorder and dementia has been seldom reported in which the clinical features are atypical and can be misdiagnosed. The main difficulty is to establish a correlation between symptoms and the cyst. When indicated, the treatment is essentially surgical and the ideal operative technique is also a matter of debate. Here, we reported a 58-year-male Chinese patient who presented with memory impairment 1 year ago. Both the physical and laboratory examinations were performed to evaluate the general conditions of the patient. Brain magnetic resonance imaging (MRI) was applied to observe SPC and the neighboring brain structures. Mini-Mental State Exam (MMSE) and Montreal Cognitive Assessment (MoCA) were used to assess cognitive function. The results of the patient’s laboratory examinations were normal. However, the patient exhibited severe sleeplessness along with cognitive deteriorations despite short-term (less than 2 weeks) use of benzodiazepines with regular dose. MRI fulfills the consensus criteria for clinical diagnosis of SPC. Furthermore, the results of MMSE and MoCA were showed mild cognitive impairment (MCI) before the treatment of SPC. After neuroendoscopic fenestration of SPC, the patient’s syndromes were disappeared, and his cognitive function was improved. In conclusion, the patient’s symptoms were due to a secondary lesion attributed to the cyst. Comprehensive clinical evaluation and MRI help diagnose SPC induced dementia.

## Introduction

The septum pellucidum develops at 10–12 weeks of gestation from the primitive lamina terminalis or the commissural plate. Its development is closely linked with that of the corpus callosum and is complete by 17 weeks of gestation (Sarwar, 1989). The septum pellucidum is lined with ependymal cells on its ventricular side whilst pia mater lines the cavity. The septum pellucidum is a thin (1.5–3.0 mm) transparent membrane that extends from the rostrum, genu and anterior portion of the body of the corpus callosum to the fornix. It separates the anterior horns of the lateral ventricles and has two layers (laminae) that are adherent to each other (Sarwar, 1989). The septum pellucidum cyst (SPC) is a cystic structure, locates between the lateral ventricles and extends between the anterior portion of the corpus callosum and the body of the fornix. Therefore, many symptoms and disease have been attributed to SPC. The descriptions of the SPC syndrome include mental disturbances, ataxia, disordered speech, epilepsy and bilateral pyramidal signs (Berti et al., 1990). SPC induced Memory disorders before operation have seldom been reported, are poorly described and then usually in association with more general impairment (von Cramon and Schuri, 1992). There is little evidence of improvement of memory impairment after the excision of SPC. Here, we first report a 58-year-male Chinese patient of SPC that showed cognitive decline and dementia with fast progress during one-year follow-up study. The patient’s physical examination was showed mild cognitive impairment (MCI) without history of stroke or depression. Furthermore, he exhibited severe sleeplessness along with cognitive deteriorations despite trials with different benzodiazepines. His laboratory examination did not show anomaly, which indicated that his general physical condition is favorable. However, magnetic resonance imaging (MRI) fulfills the consensus criteria for clinical diagnosis of SPC.

## Background

SPC, first reported in 1951, is a congenital abnormality in the midline of the brain and a sign of abnormal brain development (Ciric and Zivin, 1975; Amin, 1986). SPC is located in the midline area between the two leaflets of the septum pellucidum and is present in approximately 15% of normal adult brains (Lancon et al., 1996; Gur et al., 2013). Although SPC are usually regarded as a random finding of little clinical significance, it is considered to be a developmental anomaly which may contribute to headache or dizziness (Bodensteiner and Schaefer, 1990; Frattalone and Neely, 2011).

SPC is very rare and described as a thin vertical partition composed of two laminae, separated by a narrow cavity, fluid-containing structure, at least 10 mm interval, between the lateral ventricles (Aldur et al., 1999). However, the pathogenic factors and natural history of SPC are unknown. Most of cysts are inactive and remain asymptomatic throughout life and are discovered occasionally, but others may become symptomatic due to direct oppression on surrounding tissues and hydrocephalus by blocked cerebral spinal fluid (CSF) flow through the foramen of Monro (Hong et al., 2011). SPC with hydrocephalus has been reported occasionally, usually in single case reports or reviews. Silbert et al. (1993) have described five patients with symptoms of persistent or intermittent obstructive hydrocephalus associated with enlarged SPC. The most common presenting symptoms were intermittent headache and loss of consciousness, presumably due to sudden transient obstruction of the foramina of Monro. It is well known that the septum is part of the limbic system and that this system is involved in memory functions (von Cramon and Schuri, 1992). To date, the association of SPC with memory has seldom been described (Berti et al., 1990; von Cramon and Schuri, 1992). Here, we reported a 58-year-male Chinese patient suffering from SPC that showed cognitive decline and dementia with fast progress during 1-year follow-up study.

## Case Report

Before this study, the written informed consent was obtained from the patient, and the study was approved by the regional ethics committee of The First Affiliated Hospital of Yangtez University Medical School, and performed in accordance with the Declaration of Helsinki. The patient is a 58-year-old Chinese male who was mentally able to work as a policeman 1 year ago. He was normal nutritional and has no drug addiction habits as well as psychiatric disorders before. Six months ago, he began to suffer mild insomnia and episodes of recent memory loss, then sought medical treatment in our hospital.

His pulse was 78 beats/min and regular, and blood pressure was 130/65 mm Hg. The consciousness was sensitive and Glasgow Coma Scale sore was 15. Cranial nerves examination was negative. For motor function, the patient had normal muscle strength (grade 5) and normal muscle tone (grade 0). According to Kendall (1991) report, the grading scale of muscle strength ranges from 0 to 5. Briefly, grade 0, no visible or palpable contraction; grade 1, visible or palpable contraction with no motion; grade 2, full range of motion (ROM) gravity eliminated; grade 3, full ROM against gravity; grade 4, full ROM against gravity, moderate resistance; grade 5, normal, full ROM against gravity, maximum resistance. In terms of muscle tone, based on the Ashworth classification, muscular tone was divided into 5 grades from normal (grade 0) to limited flexion and extension (grade 4). Stance and gait was freely without involuntary movements. Finger-to-nose test, heel-knee-shin test and Romberg sign test were all negative. Sensory examination was normal. Finally, reflexes check shows symmetrical and pathologic reflexes were negative.

Laboratory examinations of routine blood, homocysteine, VitB12 and folic acid were normal. For the first time, MRI scans were not performed due to the patient’s implanted metal dentures. In order to get his MRI image, with the consent of the patient, his implanted dentures were removed before MRI scans so as to avoid inhalation of the respiratory tract and other potential hazards. His mini-mental state examination (MMSE) score was 27 (see Table 1). These results were almost normal and acceptable in this aged people.

**Table 1:** Mini-mental state examination (MMSE) scores at the patient’s first visit, second visit (worsened condition) and third visit (post-surgery with neuroendoscopic fenestration) respectively.

Items	First visit (27^th^ May, 2014)	Second visit (2^nd^ Dec, 2014)	Third visit (18^th^ Feb, 2015)
Orientation	10/10	7/10	9/10
Registration	3/3	3/3	3/3
Attention and Calculation	4/5	1/5	5/5
Recall	2/3	0/3	3/3
Language	8/9	7/9	8/9
Total	27/30	18/30	28/30

Montreal Cognitive Assessment (MoCA) screening for MCI is more sensitive than MMSE (Nasreddine et al., 2005). Then we conducted MCI exam with a serial scale for this patient. The results showed that his MoCA examination score was 25 out of 30 with mainly deficits in visuospatial/executive (4/5), delayed recall (3/5) and orientation (4/6; see Table 2). In addition, we tested Activity of Daily Living Scale (ADL) for daily life functioning, and Hamilton Anxiety and Depression Scale (HAMA, HAMD) for mental state. These results showed that memory impairments were not relevant in daily life and state of mind during his work in a police office. Therefore, we gave a diagnosis of MCI at that time according to National Institute of Neurological Disorders and Stroke-Alzheimer’s Disease and Related Disorders (NINCDS-ADRDA) Criteria 2011 (Guo et al., 2010).

**Table 2:** Montreal Cognitive Assessment (MoCA) scores at the patient’s first visit, second visit (worsened condition) and third visit (post-surgery with neuroendoscopic fenestration) respectively.

Items	First visit (27^th^ May, 2014)	Second visit (2^nd^ Dec, 2014)	Third visit (18^th^ Feb, 2015)
Visuospatial/Executive	4/5	2/5	3/5
Naming	3/3	2/3	3/3
Memory	5/5	1/5	4/5
Attention	2/2	0/2	1/2
Language	2/2	1/2	1/2
Abstraction	2/2	0/2	1/2
Delayed recall	3/5	3/5	3/5
Orientation	4/6	2/6	4/6
Total	25/30	11/30	20/30

Then, the patient was treated with alprazolam and donepezil but did not return regularly for follow-up visits. Six months after his first visit, he began to have severe sleepless and misidentification with movie characters and his family members. In addition, he could not perform simple calculations or shopping alone, even routine daily-work. His cerebrospinal fluid was collected, the pressures was 155 mm H_2_O. Bacterial and virus battery, 14–3–3, total tau, phosphorylated tau181 and Aβ42 were analyzed. All the above measurements showed negative results.

**Figure 1:**
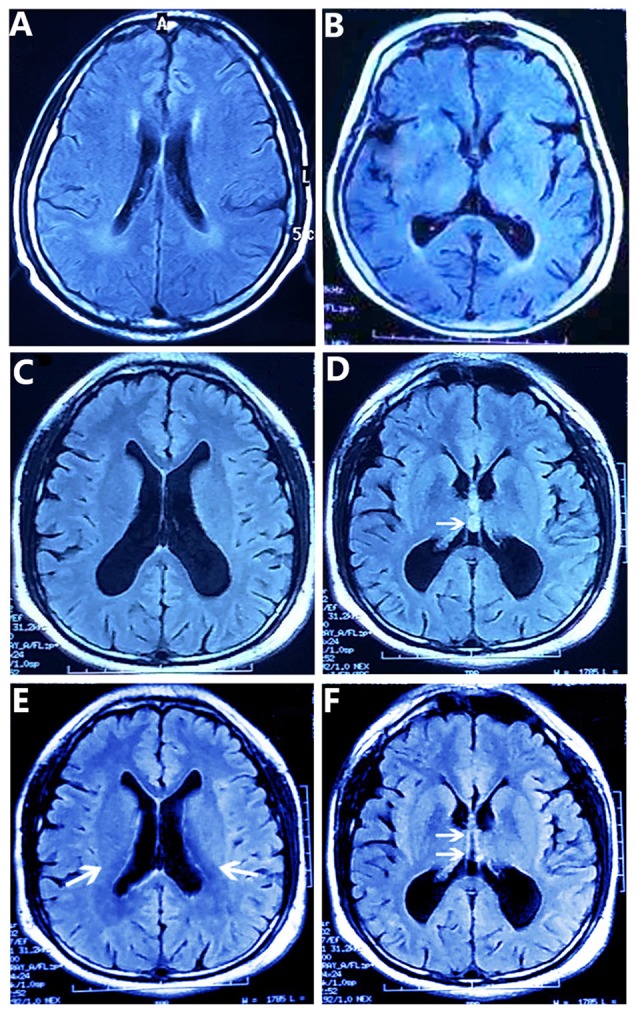
T2-weighted FLAIR MR Images obtained by magnetic resonance imaging (MRI) scans. **(A,B)** The matched control’s brain MR images show the normal lateral ventricles **(A)** and mild atrophy without septum pellucidum cyst (SPC) **(B)**. **(C,D)** The patient’s brain MR images show the enlarged double lateral ventricles **(C)** and the SPC between the lateral ventricles (arrow) **(D)**. And the walls of the cyst are laterally bowed, which compress the lateral ventricles **(D)**. After removing the SPC **(E,F)**, the patient’s brain MR images show lessened enlargement of the lateral ventricles (arrows) **(E)** and the disappeared SPC in the middle line area (arrows) **(F)**.

The patient’s MMSE score was 18 (see Table 1), therefore we gave a diagnosis of probable Alzheimer’s disease according to NINCDS-ADRDA Criteria 2011. Moreover, compared with the matched control whose brain shows mild atrophy without SPC, and his cognitive functions are normal (Figures 1A,B). The MRI results showed that the double lateral ventricles were significantly expanded (Figure 1C) and the space-occupying lesions in the midline area were clearly observed (Figure 1D). In consideration of the above tests, the final diagnosis for this patient was suspected dementia induced by SPC. Then, he was transferred to the department of neurosurgery for minimally invasive surgical treatment. Neuroendoscopic fenestration of the SPC was successfully performed via a right frontal approach using a high-resolution flexible neuroendoscopic system without complication. Communication between the cyst cavity and bilateral ventricles was constructed via a single trajectory. Two months after treatment, the patient returned us a visit. We rechecked him by MRI and MMSE. The MRI results confirmed a shrinkage of the expanded lateral ventricles (Figure 1E) and elimination of SPC (Figure 1F). Meanwhile, his MMSE score was 28 (see Table 1), and he was able to be competent at both his job and daily life. In the later 2 months of brief follow-up period, the patient showed better adherence and tolerability and no adverse events could be observed.

## Discussion

In this study, we first report the possible SPC related dementia with expanded lateral ventricle. We suppose that SPC may block CSF flow and influence the formation and maintenance of normal pressure hydrocephalus (NPH), which could exhibit associated symptoms including headache, cognitive and psychiatric disorders (Siedlecki, 2008; Lieb et al., 2015; Mongin et al., 2015). After neurosurgical treatment, the patient’s symptoms were disappeared. Based on these findings, we speculate that the reason for a progressive and reversible dementia through SPC was mainly caused by NPH. In this patient, the common symptom was acute dementia accompanied by sleepless and psychotic change. According to history of no exposure to heavy metals and toxicant, no symptoms and signs of organ failure, negative results blood and CSF test, we excluded the progressive and reversible dementia induced by bacterial and virus infection, thyroid dysfunction, autoimmune disease, and nutritional deficiency (Acosta et al., 2014). Because the symptoms resolved after surgical treatment, it is undeniable that the functions of the septum are directly associated with the occurrence of cyst. Changes in the structures of the central nervous system in the analysis of MRI before and after surgery are needed for further research on the dynamics of this anatomical area in patients with expanding cysts of SPC (Shaw and Alvord, 1997). In summary, we describe a very rare differential diagnosis of SPC induced dementia. It should be considered particularly if the symptoms are not typical for neurological dysfunctions, while the patient has gradually developed cognitive impairment. In this case, we suggest imaging test like MRI to confirm the diagnosis.

Along with the dementia-related scale battery score, the imaging results gave us enough confidence to diagnose without invasions. After having been treated with endoscopic cyst wall fenestration, MMSE score of the patient was recovered and he could return to his job. Though the treatment is minimally invasive (Cohen, 1998; Gangemi et al., 2002), further studies are needed to explore the long-term effects on his cognitive function.

## Concluding Remarks

In conclusion, this case provided a rare case with SPC induced cognitive decline and dementia featured with fast progress. Brain MRI and comprehensive clinical evaluation may help diagnose SPC and SPC induced dementia. Furthermore, cognitive decline and dementia could be attenuated after removing SPC by neuroendoscopic fenestration. We suppose that the reason for a progressive and reversible dementia may associate with expanding cyst of SPC and could be possibly caused by NPH.

## Author Contributions

L-MC and LP: study concept and design. L-MC and M-XZ: acquisition of data; analysis and interpretation of data. YW and LP: drafting of the manuscript. Y-FZ, S-HM, YY and L-XX: critical revision of the manuscript for important intellectual content. All authors read and approved the final manuscript.

## Conflict of Interest Statement

The authors declare that the research was conducted in the absence of any commercial or financial relationships that could be construed as a potential conflict of interest.

## Abbreviations

SPC: septum pellucidum cyst
MRI: magnetic resonance imaging
CSF: cerebral spinal fluid
MMSE: mini-mental state examination
MoCA: montreal cognitive assessment
MCI: mild cognitive impairment
ADL: activity of daily living scale
HAMA: hamilton anxiety scale
HAMD: hamilton depression scale
NINCDS–ADRDA: National Institute of Neurological Disorders and Stroke–Alzheimer’s Disease and Related Disorders
NPH: normal pressure hydrocephalus.
